# *Centella asiatica* and Its Metabolite Asiatic Acid: Wound Healing Effects and Therapeutic Potential

**DOI:** 10.3390/metabo13020276

**Published:** 2023-02-14

**Authors:** Lúcio Ricardo Leite Diniz, Leonardo Luiz Calado, Allana Brunna Sucupira Duarte, Damião Pergentino de Sousa

**Affiliations:** 1 National Institute of the Semiarid, Campina Grande 58434-700, Paraíba, Brazil; 2 Departament of Pharmaceutical Sciences, Federal University of Paraíba, João Pessoa 58051-970, Paraíba, Brazil

**Keywords:** natural products, medicinal plant, terpene, Apiaceae, anti-inflammatory activity, antimicrobial action, phytotherapy, scar, cicatrization

## Abstract

An intense effort has been focused on new therapeutic approaches and the development of technologies for more efficient and rapid wound healing. The research for plants used for long time in traditional medicine in the treatment of wound has become a promising strategy to obtain drugs therapeutically useful in the acute and chronic wound management. In this context, *Centella asiatica* (Apiaceae) has been used to treat a variety of skin diseases, such as leprosy, lupus, varicose ulcers, eczema and psoriasis, in Asiatic traditional medicine for thousands of years. Studies have shown that *Centella asiatica* extracts (CAE) display activity in tissue regeneration, cell migration and wound repair process by promoting fibroblast proliferation and collagen synthesis. Preliminary findings have shown that the asiatic acid is one of the main active constituents of *C. asiatica*, directly associated with its healing activity. Thus, this study discusses aspects of the effects of *Centella asiatica* and its active component, asiatic acid, in different stages of the healing process of cutaneous wounds, including phytochemical and antimicrobial aspects that contribute to its therapeutic potential.

## 1. Introduction

### 1.1. Skin and Wound Healing

The skin is a specialized organ comprised of three different structural and functional layers. The epidermis, the outermost layer, is avascular, impermeable and composed by dead cells, immune system cells, melanocytes, sebaceous glands, sweat glands, and hair follicles. The dermis, the middle layer, is rich in extracellular matrix (ECM), vasculature, mechanoreceptors, connective tissue, nerve endings hair follicles and glands. The subcutaneous tissue, the innermost layer, is mostly made up of fat, connective tissues, larger blood vessels and nerves [1]. The skin provides many pivotal homeostatic functions, including regulating thermostability and fluid balance. Moreover, the skin is primary body defense barrier, protecting the internal structures against infections and physical, mechanical and chemical damages. Thus, the maintenance of its structure and function is critical for survival [1,2]. Skin integrity is restored by a physiological process aimed at repairing the damaged tissues, which is dependent on many cell types and mediators interacting in a highly sophisticated temporal and overlapping sequence [3,4,5]. Based on the pathogenesis and consequences, skin wounds might be divided into acute and chronic wounds. Acute wounds undergo a series of molecular events that eventually result in the regaining of structural integrity. By contrast, chronic wounds fail to resolve and are characterized by pathologic processes, such as continuous inflammation, persistent infections and necrosis [2,3,4,5,6]. It is critical to know the main molecular and cellular inflammatory mediators in understanding skin healing progression for designing targeted and effective therapies. In general, acute wound proceeds in four overlapping phases: hemostasis, inflammation, proliferation and remodeling [3,4,5,6].

The wound repair begins with hemostasis, where a platelet plug prevents blood loss and a preliminary fibrin matrix are formed. Platelets also produce platelet-derived growth factor that along with bacterial products attract inflammatory cells to the site of injury. The inflammatory phase happens during the first 24 h post injury and remains intensive for 2–5 days unless the wound gets infected. This phase is essential for supplying growth factor and cytokine signals that are responsible for cell and tissue movements with subsequent development of wound repair mechanisms [2,7]. The inflammation is established with the neutrophil influx, which is promoted by histamine release from mast cells. The neutrophils promote release of pro-inflammatory cytokines secretion (e.g., TNF-α, IL-1β and IL-6), phagocytosis and protease secretion, contributing for others inflammatory cells attraction, amplification of the inflammatory response, microbial pathogens deaths and stimulation of the regenerative and remodeling factors [8,9]. The macrophages play a fundamental role in the resolution of inflammation through the intense phagocytic activity and secretion of pro-inflammatory cytokines, such as IL-1, IL-6, and TNF-α, which act on key events of inflammation to promote the return to homeostasis. During early phase of inflammation, the production of inflammatory mediators promotes leukocyte accumulation and survival in the inflammatory site, creating a favorable environment for the resolution phase leading to return to tissue homeostasis. For example, IL-1β and TNF-α, two cytokines required for activating the inflammatory response, mediating the recruitment, activation, and adherence of circulating phagocytic cells, might also triggered an anti-inflammatory cascade resulting in the production of IL-10, an anti-inflammatory cytokine that maintains the balance of the immune response, allowing the clearance of infection while minimizing damage to the host [6,7,8]. It is also observed the synthesis of numerous potent growth factors (e.g., TGF-β, PDGF and VEGF), which promote cell proliferation and tissue restoration following injury. In addition, inflammatory cells activate cyclooxygenase-2 (COX-2), which in turn catalyses the conversion of arachidonic acid to prostaglandin E2 (PGE2) and other pro-inflammatory mediators. PGE2 plays a role in the swelling, redness, and pain in the wound area [2,9]. In general, the amount of inflammation determines the extent of scar formation [3,10]. It is noteworthy that the total inhibition of the inflammatory response during acute wound might also promote delay and impairment in the development of subsequent stages of wound healing. In contrast, an intense or persistent inflammation might produce serious cutaneous damages and loss of function, as observed in chronic wound [4,5]. The wounds that take longer than 12 weeks after injury to heal are termed chronic wounds [11]. The non-healing wounds remain to be one of the most complications of various diseases such as diabetes, cancer and venous ulcer, increasing the vulnerability to the invasion of microorganism in the injured tissue and their subsequent consequences [11,12]. Unhealed wounds are associated with increases of morbidity, loss of function and worsening quality of life. Chronic wounds affect 6.5 million people in the United States, costing the healthcare system an estimated 50 billion dollars annually [12,13,14]. Thus, a therapeutic agent with significant anti-inflammatory activity might be also a relevant tool in the treatment of chronic wounds.

The dynamic progression from the inflammatory phase to the proliferative phase is critical for the effective wound healing process. The proliferative phase occurs approximately 3–10 days after wounding and it is characterized by formatting granulation tissue, neovascularization, re-epithelialization, and immunomodulation [3,7]. The granulation tissue is the hallmark of the proliferative phase and it is formed by new capillaries, collagen and activated fibroblasts that synthesize new extracellular matrix (ECM), which in turn provides a dynamic scaffold for cell migration, adhesion, proliferation, differentiation, and maturation as well as help contract the wound [6,9]. During the proliferation, keratinocytes migrate to close the wound gap, blood vessels reform through angiogenesis, and fibroblasts replace the initial fibrin clot with granulation tissue. Macrophages and regulatory T cells (Tregs) are also vital for this stage of healing. Neovascularization provides the delivery of nutrients and maintenance of oxygen homeostasis, to allow cellular proliferation and tissue regeneration to occur [4,5,6,7,8,9,10].

Remodeling is the last phase of acute wound healing and occurs at, approximately, from day 10 to up to 100 days after injury. This phase starts by the end of the proliferation phase with the formation of granulation tissue and reepithelization through keratinocytes and ECM deposition by the fibroblasts and endothelial cells. The mechanical tension and increased inflammatory cytokines concentration, like TGF-β, drive fibroblast differentiation into myofibroblast, which attaches to collagen and promotes wound contraction and scar maturation [6,7,10]. Furthermore, the myofibroblasts also produce substantially more collagen than their regular counterparts. During the maturation of the wound, the collagen III in the ECM, produced in the proliferative phase, is replaced by the stronger collagen I, which has higher tensile strength but takes longer to deposit as well as to improve the tensile strength, wound contraction and breach power [10,15,16].

### 1.2. Wound Healing and Phytomedicine

Every year, nearly one billion people suffer from acute and chronic cutaneous wounds in response to skin injuries caused by microbial infections, extreme temperatures, ultraviolet radiation, skin diseases, surgery, trauma, or burns with significant undesired physical and psychological effects [3,5]. Acute and chronic wounds are common health problems and greatly affect both individual patients and the healthcare system, creating a negative impact on the economy worldwide. For example, in the United States alone, the overall costs associated with chronic wounds are approximated to be $50 billion, scars from surgical incisions and trauma account for nearly $12 billion, and burns account for $7.5 billion USD in healthcare costs per year [12,13,14]. Despite some therapies are used in the wound management, including changes of systemic and lifestyle factors, surgical removal of necrotic tissue, antibacterial and anti-inflammatory drugs, skin dressing, skin substitutes, negative pressure therapy and others treatments, the results are still unsatisfactory [17,18]. There is a clinically unmet need to novel therapies that are financial, physiological and practical viable for the wound care setting. In this context, herbs used for treatment of skin wounds in folk medicines around the world have become the focus of the scientific researchers for new therapeutic agents, which can act in single or multiple mechanisms in any one or more individual phases of the wound repair process [17,18]. In fact, various studies provide evidence that many plants used in traditional medicine, generally topically, for treating wounds is a rich source of research for new compounds that improve the overall outcome of the cutaneous wounds closure. For example, it has reported that the treatment with essential oils extracted from *Rosmarinus officinalis*, *Aloe vera*, *Calendula officinalis* and *Croton zenhtneri* extracts act in different targets of one or more phases of wound healing, including activation of transcription factors, increase of anti-inflammatory mediators and reduction of pro-inflammatory mediators during the inflammatory phase [19,20,21,22,23,24]. In proliferative and remodeling phases, some extracts have promoted regeneration of granulation tissue, angiogenesis and collagen deposition, accelerating the wound healing [19,20].

### 1.3. Centella Asiatica and Asiatic Acid

*Centella asiatica* (L.) Urban is a small perennial herbaceous plant, belonging to the Apiaceae family and widely spread in the Southeast Asian countries, such Malaysia and Indonesia, where it is commonly known as Gotu kola, mandukparni, Indian pennywort, jalbrahmi and pegaga nyonya [25,26]. It has been used to treat a variety of diseases in asiatic traditional medicine for thousands of years, as suggested by the reports of its presence in the list of medicines of “miracle elixirs of life” and “Sushruta Samhita”, historic medicine texts, dated over 2000 years ago, from traditional Chinese and Indian medicines, respectively [17,27]. It is an herb also used in traditional medicine from Sri Lanka, Java and other Indonesian islands [25,26,27,28,29,30]. The Indian pharmacopoeia recommended *Centella asiatica* for the treatment of various skin conditions such as leprosy, lupus, varicose ulcers, eczema and psoriasis [30,31,32]. Flavonoids, plant sterols, eugenol and pentacyclic triterpenoids are among the several interesting phytochemical constituents found in the *C. asiatica* [25,26,33]. Most of studies have reported the asiatic acid, as one of the main active constituents producing the wound healing activity of *C. asiatica* [33,34].

**Figure 1:**
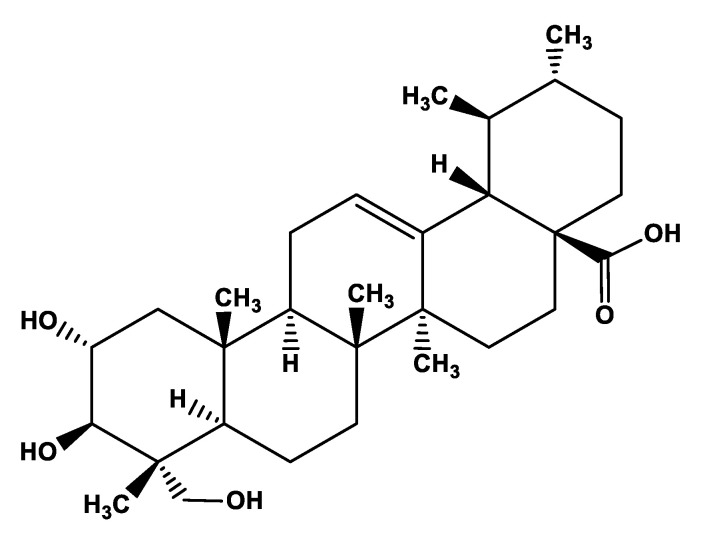
Chemical structure of asiatic acid.

The asiatic acid (ac), an aglycone ursane type pentacyclic triterpene saponin (C_30_H_48_O_5_), is one of the main chemical components of *Centella asiatica* and it plays pivotal role in wound healing activity of *C. asiatica* [35,36,37,38]. Pharmacokinetic data show that asiatic acid has poor bioavailability, hardly soluble in water (5.98 × 10^−2^ mg/L at 25 °C). It is absorbed in the jejunum and distributed in many tissues (e.g., plasma, brain, heart, liver, kidney, colon, and bladder) by binding with albumin and mainly metabolized in the intestine [33]. It is also known that the chemical modification of the asiatic acid’s backbone improved its bioavailability and biological activity [34,35,36,37,38,39,40,41]. Asiatic acid is the most potent component of *C. asiatica* for induction of expression of TNFAIP6, a hyaladherin involved in extracellular matrix remodeling and modulation of inflammation, in human fibroblasts [42]. Asiatic acid has also been found to increase collagen synthesis, which is important in the healing process [43,44,45]. Therefore, the present study discusses the effects of *Centella asiatica* and its active component, asiatic acid, at different stages of the healing process of cutaneous wounds. The molecular structure of asiatic acid is given in Figure 1.

## 2. Materials and Methods

The present review was carried out based on a survey of the literature on *Centella asiatica*, asiatic acid and wound healing. The search, performed in the PubMed database, included studies published until December 2022 and used the following keywords: *Centella asiatica*, asiatic acid, wounds, wound healing, skin wounds, cutaneous wound, skin repair and cutaneous repair. Only reported data of wound healing effects of isolated *Centella asiatica* extracts and asiatic acid assessed by in vitro assays or experimental models of wound healing was selected. Results obtained from others *C. asiatica*’s constituents well as its combination with other bioactive drugs was not considered. Only the scientific publications published in the English language were selected.

## 3. *Centella asiatica* and Cutaneous Repair Process

**Figure 2:**
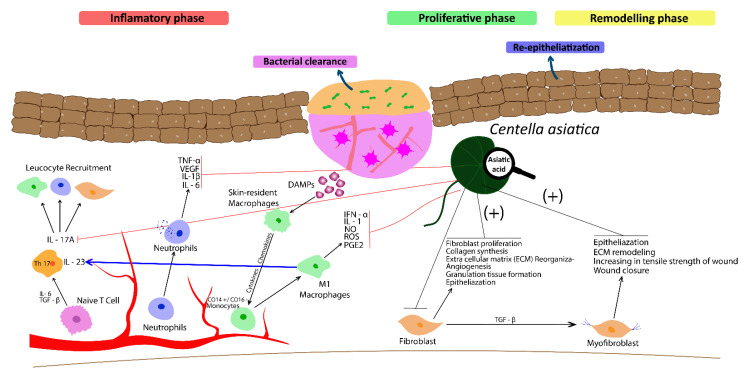
*Centella asiatica* extract (CAE) and its bioactive constituent, asiatic acid (Aa) display a decrease in the wound area and faster healing by increasing collagen synthesis, and cellular proliferation, fibroblast division and re-epithelialization during proliferative and remodeling phases of wound healing. CAE and Aa also act in the inflammatory phase by inhibiting recruitment of immune defense cells, reducing synthesis of pro-inflammatory cytokines (TNF-α, IL-6, and and IL-1β) and growth factors (TGF-β, PDGF and VEGF). Moreover, Aa inhibits the increase of serum levels of IL-17/IL-23. This figure was created with Adobe Illustrator.

Experimental and preclinical studies have been increasingly supportive for traditional use of the *Centella asiatica* extracts (CAE) for wound healing. Although phases of wound healing are overlapped and connected, the current review presents the effect of CAE and asiatic acid (Aa) on relevant cellular and molecular mechanisms that characterize each stage of the wound healing process, in an attempt to facilitate the understanding of the underlying mechanism involved in wound healing activity of both *C. asiatica* and asiatic acid. The Figure 2 and Table 1 illustrate the main effects of both CAE and Aa in phases of cutaneous repair and regeneration process.

### 3.1. The Effect of Centella asiatica and Asiatic Acid in the Inflammatory Phase of Wound Healing

Despite the reduced number of studies with focus on the inflammatory phase when compared to another phase of wound healing, the anti-inflammatory activity of CAE and Aa might be strongly evidenced in the inflammatory skin diseases models, such as psoriasis and atopic dermatitis [30,31,41]. Experimental studies performed in different animals models of wound healing, including phthalic anhydride (PA)-induced atopic dermatitis, excision, incision, burn and diabetic wounds models, have shown that the topical and oral administration of CAE reduces massive neutrophil recruitment accompanied by reducing release of TNF-α, IL-1β, IL-6, and IgE, and inhibition of the expression of iNOS, COX-2, NF-κB and lipoxygenase (LOX) activity in the site of skin injury [31,32,41,46,47,48,49,50,51,52]. Recently, Kukula et al. [53] showed that asiatic acid treatment (100 mg/kg, p.o) suppressed the imiquimod-induced increase in serum levels of IL-17A and IL-23 in the imiquimod-induced psoriasis model [53]. Studies have demonstrated that IL-23 induces the differentiation of naive CD4^+^ T cells into highly pathogenic helper T cells (Th17/ThIL-17) that produce IL-17, IL-17F, IL-6, and TNF-α [2,5,6].

### 3.2. The Effect of Centella asiatica and Asiatic Acid in the Proliferative Phase of Acute Wound Healing

The experimental and clinical findings have suggested that many of the beneficial effects on cutaneous repair process produced by *C. asiatica* are directly associated with their actions on the proliferative phase of the wound healing process, such as stimulation of fibronectin and collagen synthesis, maintenance of connective tissue and the strengthening of weakened veins [27,28,29,30,31,32,33,34,35,36,37,38,39,40,41,42,43,44,45,46,47]. Asiatic acid has been reported to possess wound healing activity by increasing collagen formation and angiogenesis. Apart from showing a stimulation of the collagen synthesis in different cell types, the asiatic acid was shown to increase the tensile strength of the newly formed skin, furthering the healing of the wounds [38,39,40,41]. Over the last decades, diverse in vitro studies performed with human dermal fibroblast showed that both aqueous and alcoholic extracts of *Centella asiatica* as well as asiatic acid increase type I collagen and fibronectin synthesis [43,44]. In agreement, different in vivo studies have reported that *C. asiatica* extracts (CAE) and asiatic acid (Aa) increase fibroblast proliferation, collagen synthesis and angiogenesis [35,36,37,38]. Some researches performed in incision models of skin wounds reported that the oral treatment with CAE promoted a decrease in the wound area and faster healing by increasing collagen synthesis, cellular proliferation, fibroblast division and reepithelialization [40,41,42,43,44,45,46]. In 1996, Suguna et al. [54] showed that the oral and topical administration of an alcoholic extract of *C. asiatica* increased cellular proliferation, protein and collagen content of granulation tissues on rat incision model [54]. Moreover, in guinea pig punch wounds topical applications of 0.2% solution of asiaticoside produced a significant increase of hydroxyproline, in tensile strength, collagen content and better epithelization [55]. Sunilkumar et al. [56] showed that the topical formulation of CAE applied 3 times daily for 24 days to open wounds in rats increased cellular proliferation and collagen synthesis at the wound site, leading to wounds epithelialized faster and higher the rate of wound contraction when compared to untreated control wounds [56]. In accordance, Shetty et al. [57] showed that ethanolic extract of the leaves from *C. asiatica* enhanced wound breaking strength, granulation tissue weights, granulation tissue breaking stretch with rate of wound contraction faster than the controls in the incision wound healing model. In addition, CAE attenuated the wound-healing suppressing action of dexamethasone in a rat model [57].

### 3.3. The Effect of Centella asiatica and Asiatic Acid in Remodelling Phase of Acute Wound Healing

Studies have shown that both *Centella asiatica* and asiatic acid act on cellular and molecular mechanisms of the remodelling phase [31,32]. Studies assessed in human dermal fibroblast culture reported an increase of type I collagen synthesis and crosslinking promoted by *C. asiatica* [43,44]. The oral and topical treatment with different pharmaceutical formulation containing CAE and/or Aa has been found to be efficacious in the improvement of remodelling stage of wound healing, through stimulation of extracellular matrix accumulation, maintenance of granulation tissue, increase of synthesis, maturation and the cross-linking of collagen, and high tensile strength of the newly formed skin, [45,58,59,60,61,62,63,64,65,66]. In 2012, Wu et al. [39] investigated the action pattern and mechanisms of active constituents of *C. asiatica* herbs on burn wound healing via both in vitro and in vivo assay. According to obtained results, authors drew a conclusion that the wound-healing effect promoted by *C. asiatica* and its bioactive glycosides is probably through activating TGF-β/Smad pathway, as evidenced by elevated p-Smad 3 level, raised TGF-β 1 and TβRII mRNA levels and downregulated Smad 7 expression, in fibroblast. In addition, it was also observed an elevated procollagen type I and type III expression both at mRNA and protein levels in *C. asiatica* glycosides-treated groups [39]. These data are in agreement with the preliminary findings that triterpenes of *C. asiatica* produce stimulation of TGF-β 1, fibroblasts proliferate and secrete extracellular matrix (ECM), mainly collagen type I and type III, and also partially differentiate into myofibroblasts, which in turn contribute to an accelerated closure by contraction in remodeling phase of wound healing [44,45,59]. Yao et al. [66] showed that gelatin nanofibres containing CAE promoted an increase of fibroblast proliferation and collagen synthesis. The wound areas of rat skin treated presented the highest recovery rate compared with those treated with gauze, neat gelatin membranes and commercial wound dressings [66].

### 3.4. The Effect of Centella asiatica and Asiatic Acid in Chronic Wound Healing

Even though the wound healing effects of CAE and its bioactive constituents on chronic wounds have not been intensely investigated and the published data are still scarce and unsatisfactory, there are some evidence of promising therapeutic use of *C. asiatica* in treatment of chronic wounds [67,68,69,70]. In streptozotocin-diabetic rats, the topical application of a solution containing asiatic acid over punch wounds increased hydroxyproline content, tensile strength, collagen content, as well as quicker and better maturation and cross linking of collagen and epithelization thereby facilitating the healing of diabetic rats, where healing is delayed [67]. Moreover, both CEA and asiatic acid display significant anti-inflammatory activity and consequence might be an important tool to diminish the exacerbated inflammation found in chronic wounds. As mentioned above, asiatic acid treatment (100 mg/kg, p.o) suppressed the imiquimod-induced increase in serum levels of IL-17A and IL-23, an important inflammatory pathway found in the pathogenesis of some chronic cutaneous diseases [53]. Paocharoen [49] reported that the treatment with CAE capsule, three times a day (50 mg of extracted asiaticosides), was effective in the wound healing promotion and also suppress the scar in diabetic wound patients (Table 1). Moreover, it was not observed serious side effect of the CEA capsule group [49]. Other research studies have proven that oral and/or topical CAE improves skin disorders associated with wound healing impairment and chronic wounds. For example, in a study performed to evaluate the efficacy of the oral and topical CAE in 159 Type 2 Diabetes Melitus patients with dry skin, Legiawati et al. [68] related that the oral and topical CAE combination improved dry skin condition through increasing superoxide dismutase (SOD) activity in diabetic patients with controlled blood glucose [68], Figure 2. Previously, Kuo et al. [69] observed that CA cream for 14 days improved diabetic foot ulcer [69]. Jenwitheesuk et al. [70] reported that 7% of *Centella asiatica* cream application in split-thickness skin graft (STSG) patients showed better scar development [70]. In recent study carried out by Liu et al. [67], it was observed that 8% *Centella asiatica* total glycoside accelerated the healing speed of diabetic cutaneous ulcer wounds, a chronic and refractory complication of diabetes mellitus [67].

**Table 1:** Wound healing effects of *Centella asiatica* and asiatic acid in different phases of skin repair process.

Inflammatory Phase of Acute Wound Healing			
Experimental Model	Wound Healing Effect	Cellular and Molecular Mechanism	**Reference**
Excision Wounds	CAE complexed with HP-β-CD healed completely the excising wound in rats after 14 days.	The authors attributed the wound healing effect of CAE to presence of asiaticoside that stimulates keratinization, increases the tensile strength and synthesis of collagen and inhibits the inflammatory phase.	[37]
Phthalic anhydride (PA)-induced atopic dermatitis	CAE attenuated the development of PA-induced atopic dermatitis.	CAE (1, 2, 5 µg/mL) inhibited mast cells and infiltration of inflammatory cells, expression of iNOS and COX-2, and NF-κB activity as well as the release of TNF-α, IL-1β, IL-6, and IgE. In addition, CAE potently inhibited NF-κB DNA binding activities in RAW264.7macrophage cells.	[47]
Excision Wounds	CAE reduced the wound area and wound healing period of full-thickness wounds	CAE (100 mg/kg) increase the NOS activity and the levels of TGF-β.	[50]
Imiquimod-induced psoriasis	Asiatic acid reduced imiquimod-induced inflammation	Asiatic acid (100 mg/kg) inhibited the increase in serum levels of IL-17A and IL-23 induced by imiquimod	[53]
Incision, Burn and Diabetic wounds	CAE and Aa reduced the inflammation and accelerated the wound healing	CAE and Aa reduced inflammatory cells recruitment and reduced pro-inflammatory (e.g., TNF-α, IL-1β and IL-6) levels.	[48,52]
Proliferative phase of acute wound healing			
Human Fibroblast cells	CAE and Aa promoted granulation tissue formation and increased the tensile strength	CAE and Aa stimulated fibronectin and collagen synthesis	[58,61]
Excision and Incision Wound	CAE and Aa promoted a decrease in the wound area and faster healing of excision wound in rats	CAE and Aa increased collagen synthesis and fibroblast proliferation	[37,39]
Incision Wound	CAE accelerated the wound healing of rat incision model	CAE increased cellular proliferation, protein and collagen content of granulation tissues	[54]
Open wound	The topical formulation of CAE applied 3 times daily for 24 days wounds promoted epithelialized faster and higher the rate of wound contraction to open wounds in rats	CAE increased cellular proliferation and collagen synthesis	[56]
Dexamethasone-suppressed incision wound	Animals treated with CAE showed faster wound contraction than untreated animals	CAE enhanced wound breaking strength, granulation tissue weights, granulation tissue breaking stretch	[57]
Remodelling phase of acute wound healing			
Human Fibroblast	Aa induced collagen I synthesis	Aa induced human collagen I synthesis through TGFβ receptor I kinase (TβRI kinase)-independent Smad signaling	[43]
Burn wound	Aa decreased wound area and faster healing	*C. asiatica* and its bioactive glycoside raised TGF-β 1, TβRII and procollagen type I and type III expression	[39]
Tongue wounds	CAE increase wound contraction and faster oral tissue regeneration on the healing process	*C. asiatica* was effective to promote collagen deposition and extracellular matrix accumulation	[66]
Incision Wound	CAE and Aa accelerated the wound healing process	CAE and Aa stimulated extracellular matrix accumulation, maintenance of granulation tissue, increase of collagen synthesis and tensile strength force	[45,60]
Chronic wound healing			
Streptozotocin-induced diabetes	As facilitated the healing process of diabetic rats	Asiatic acid increased hydroxyproline content, tensile strength, collagen content, maturation and cross linking of collagen and epithelization	[67]
Type 2 diabetic patients	CAE, was effective in the wound healing promotion and suppress the scar in diabetic wound patients.	_	[49,67,68]

## 4. *Centella asiatica* Antimicrobial Activity

Medicinal plants are rich in bioactive compounds that have various antimicrobial properties [71]. In this context, Jayaprakash and Nagarajan [72] evaluated the antimicrobial potential of extracts obtained from the leaves of *Centella asiatica*. The crude extracts of acetone and methanol were highly active compared to the extract obtained using petroleum ether. The methanolic extract showed activity against clinical bacterial strains of *Streptococcus faecalis*, *Streptococcus pyogenes*, *Escherichia coli* and an inhibition zone of 11 mm against *Staphylococcus aureus*. In another study, the methanolic extract of *C. asiatica* showed greater inhibitory activity than acetone, ethyl acetate and aqueous extracts against different bacteria [73]. These findings are similar to reported that the methanolic extract of *C. asiatica* has antimicrobial activity against both gram-positive *S. aureus* and methicillin-resistant *S. aureus* (MRSA) [74]. The methanolic extract of *C. asiatica* demonstrated promising antibiofilm activity against *V. cholerae* [75]. The dichloromethane: methanol extract of *C. asiatica* showed an inhibition of *S. aureus*, *E. coli*, *S. typhi*, *B. subtilis* and *Shigella sonnei* [76]. Dhinam et al. [77] observed that methanol and ethanol extracts of *C. asiatica* exhibited maximum zone of inhibition against all microorganisms tested. According to Aftab et al. [78] secondary metabolites such as alkaloids, terpenoids and tannins are extracted in solvents with higher polarity such as methanol. So, the antimicrobial activity of extracts using this solvent can be attributed to the presence of these compounds [78]. Mudalina [79] found that the ethanolic extract of *C. asiatica* has antimicrobial activity against Mycobacterium tuberculosis H37Rv, *Escherichia coli*, *Staphylococcus aureus* and *Salmonella typhi*. However, in a study previously developed by Sultan et al. [80] the ethanol extract *C. asiatica* did not show any antimicrobial activity against *Staphylococcus aureus* and *Escherichia coli*. Extracts obtained from the root, stem and leaves of *C. asiatica* using ethanol, chloroform and petroleum ether, exhibited antimicrobial activity against *Escherichia coli*, *Staphylococcus aureus*, *Aspergillus niger* and *Rhizopus stolonifer*. Among the three extracts used in the study, the ethanol extracts showed the greatest inhibition [71]. Likewise, Idris and Nadzir [81] observed that the ethanolic extract of *C. asiatica* showed the highest antimicrobial activity followed by methanol and aqueous extracts for *Aspergillus niger* and *Bacillus subtilis* by the disk diffusion method. The ethanolic extract *of C. asiatica* also showed antimicrobial activity against *C. albicans* (MIC= 7.81), *Aspergillus niger* (MIC = 125 mg/mL), *S. aureus* (MBC= 15.63 mg/mL), *S. typhimurium* (MBC = 15.63 mg/mL), and *E. coli* O157:H7 (MBC = 62.50 mg/mL) [82]. Nasution et al. [83] evaluated the antimicrobial activity of ethanolic, aqueous and chloroform extracts of *C. asiatica* leaf and root against *Escherichia coli*, *Staphylococcus aureus*, *Staphylococcus albus*, *Streptococcus pyogenes*, *Pseudomonas aeruginosa*, *Streptococcus pneumoniae*, *Aspergillus niger*, *Aspergillus flavus*, *Microsporum boulardii* and *Candida albicans*. Particularly, ethanol was the best extractive solvent for antimicrobial properties of *C. asiatica* leaf and root followed in order by chloroform and water. The ethanolic extract of *C. asiatica* root is more effective as an antifungal agent than the ethanolic extract of *C. asiatica* leaf, however, the effectiveness of the ethanolic extract of *C. asiatica* leaves as an antibacterial is much better than the ethanolic extract obtained of the root of this plant. This result can be explained by the concentration of bioactives that varies from one part of the plant to another [71,83]. *Bacillus subtilis*, *Staphylococcus aureus*, *Escherichia coli*, *Pseudomonas aeruginosa* and *Shigella sonnei* were selected to study the antibacterial activity of *C. asiatica* essential oil. The essential oil showed broad-spectrum antimicrobial activity against all organisms tested with MIC values ranging from 1.25 to 0.039 mg/mL [84]. In contrast, Paudel et al. [85] evaluated the antimicrobial activity of the essential oil of the aerial parts of *C. asiatica* and it did not show bioactivity against *Bacillus cereus*, *Staphylococcus aureus*, *Escherichia coli*, *Pseudomonas aeruginosa* and *Aspergillus niger*. Recently, Khan et al. [86] also verified the activity of extracts of *C. asiatica* leaves and in-vitro leaf-calli using solvents methanol and ethyl acetate and observed that extracts showed antibacterial activity against *C. violaceum* 12742 and *P. aeruginosa* PAO1.

## 5. Phytochemistry of *Centella asiatica*

The medicinal properties of *C. asiatica* as antidiabetic, antimicrobial, antidiuretic, antioxidant, anti-inflammatory, and cardioprotective are directly related to the production and concentration of several secondary metabolites [87,88]. Among these compounds, triterpene saponins are the main metabolites, which have been confirmed to be responsible for the biological activity of *C. asiatica* [89,90]. In this context, madecassoside is a major pentacyclic triterpene saponin from *C. asiatica*, with diverse pharmacological activities and widely used in medicines, food and cosmetics [90]. Recently, Ren et al. [89], isolated and purified two new ursane-type triterpene saponins, named asiaticoside H (1) and I (2) from the whole plants of *C. asiatica* [89]. Furthermore, a phytochemical investigation of the commercial extract of *C. asiatica* leaves allowed the characterization and quantification of four secondary metabolites including ten polyphenols and fourteen ursane- or oleanane-type triterpenoids in the sapogenin or saponin form and resulted in the discovery of new triglycoside saponin of the unprecedented 2α,3β,6β,23-tetrahydroxyolean-13(18)-en-28-oic acid (isoterminolic acid) [91]. Jayaprakash and Nagarajan [72] revealed the presence of alkaloids and flavonoids in extracts of *C. asiatica* using the solvents petroleum ether, acetone and methanol. Saponins, phenols, steroids, glycosides, tannins, terpenoids and triterpenoids are absent in petroleum ether extracts and present in acetone and methanol extracts. Furthermore, cardiac glycosides and resins are absent in all extracts [72]. However, the study by Sieberi et al. [76] determined the presence of cardiac glycosides in the dichloromethane: methanol extract of *C. asiatica*. The phytochemical screening of this extract determined the presence of alkaloids, flavonoids, phenolics, terpenoids, cardiac glycosides, saponins, steroids and tannins [76]. The phytochemical evaluation of *C. asiatica* at different harvest time intervals revealed the presence of phenols, flavonoids, flavonones, tannins, alkaloids, steroids and carbohydrates. Samples collected in the first harvest showed higher amounts of phytoconstituents and a higher content of asiatic acid [92]. Another seasonal assessment of triterpenes and phenolic compounds in *C. asiatica* revealed that the collection of *C. asiatica* in summer returns the highest yield of the target triterpenoids, kaempferol and chlorogenic acid [93]. Polash et al. [87], evaluated the phytochemical content of the ethanol extract of the leaf and stem of *C. asiatica*. The leaf extract showed a higher presence of flavonoids when compared to the stem extract, the phenol content was higher in the stem extract when compared to the leaf extract and the leaf extract showed a maximum amount of tannin, saponin and glycoside. In contrast, there was no saponin present in the stem extract of *C. asiatica* [87]. Dewi and Maryani [94] detected kaempferol and quercetin in the ethyl acetate extract of this plant [94]. Several chromatographic methods have been documented to evaluate the chemical composition of *C. asiatica* [91,95,96,97]. The methods of analysis gas chromatography-mass spectrometry (GC-MS) and liquid chromatography-mass spectrometry (LC-MS) identified the phytocomponents methyl pyromeconic acid, methoxy vinyl phenol, 3′,5′-dimethoxyacetophenone, 3′,5′-dimethoxyacetophenone, beta-D-ribofuranoside, cyclohexanecarboxylic acid, 5-methoxy-2,2,8,8-tetramethyl-acetate, Nobiletin, maltol, 3′,5′-dimethoxyacetophenone, papyriogenin A, asiatic acid, asiaticoside, madecassoside and madecassic acid [96]. In the study developed by Ondeko et al. [98] the LC-MS and GC-MS analyzes of the methanolic extracts identified 22 and 33 compounds, respectively. Rumalla et al. [99] identified a ursane-derived saponin (23-O-acetylmadecassoside) and an oleanane-derived saponin (23-O-acetylasiaticoside B). The main bioactives found by Bhuyar et al. (2020) [100] in the leaves, stems and roots of *C. asiatica* were n-hexadecanoic acid (99%), *cis*-vaccenic acid (91%), 5-hydroxymethylfurfural (88%) and tetradecanoic acid (86%). The essential oil of the aerial parts of *C. asiatica* contains eleven monoterpenoid hydrocarbons (20.20%), nine oxygenated monoterpenoids (5.46%), fourteen sesquiterpenoid hydrocarbons (68.80%), five oxygenated sesquiterpenoids (3.90%), and one sulfide sesquiterpenoid (0.76%). α-Humulene (21.06%), β-caryophyllene (19.08%), bicyclogermacrene (11.22%), germacrene B (6.29%), and myrcene (6.55%) were the predominant constitutes [84]. *C. asiatica* also has several constituents such as triterpenoid saponins, asiatic acid, madecassic acid, terminolic acid, vanillic acid, succinic acid, asiaticoside, asiaticoside-B, madecassoside, asiaticodiglicoside [78,95,101]. The analysis of the plant extract of *C. asiatica* leaves showed the presence of bioactive compounds, for example proanthocyanin (11.964 µg/g), flav-3-ol (2.5900 µg/g), spartein (3.0122 µg/g), flavonones (2.1836 µg/g), kaempferol (0.7273 µg/g), naringenin (2.7523 µg/g), resveratrol (10.8596 µg/g), tannin (4.4377 µg/g), rutin (11.8883 µg/g), quinine (10.4490 µg/g), and ribalinidine (3.0500 µg/g) [88].

## 6. Antimicrobial Activity of Asiatic Acid

Asiatic acid exhibits a variety of antimicrobial activities and showed substantial inhibitory effects in nineteen *Clostridium difficile* isolates collected from different sources with minimum inhibitory concentrations ranging from 10 to 20 μg/mL. This metabolite induced membrane damage and alterations in the morphological ultrastructure in *C. difficile*, thus causing the extravasation of intracellular substances. Furthermore, it also exhibited an inhibitory effect on cell motility, but did not interfere with biofilm formation and spore germination [102]. Asiatic acid also showed antifungal activity against eight strains of *C. albicans* and could work synergistically with fluconazole against resistant *C. albicans* [103]. Sun et al. [104] investigated the antimicrobial potential of asiatic acid extracted from pomegranate peels. The authors showed that asiatic acid exhibited selective inhibitory activity against *Staphylococcus aureus* with a minimum inhibitory concentration (MIC) of 16 μg/mL. Asiatic acid showed in vitro anti-mycobacterial activity against *Mycobacterium tuberculosis* H37Ra, *Mycobacterium bovis* BCG and *Mycobacterium smegmatis* [105]. Bacteria causing urinary tract infections such as *Escherichia coli*, *Enterobacter cloacae* and *Pseudomonas aeruginosa* showed a decrease in survival and a weakened ability to create biofilms demonstrating significant effects of asiatic acid on bacterial cell survival [106]. Previously, this compound also increased the susceptibility of *Pseudomonas aeruginosa* biofilms to tobramycin [107]. The effects of asiatic acid on food-borne bacteria *Escherichia coli* O157:H7, *Salmonella Typhimurium* DT104, *Pseudomonas aeruginosa*, *Listeria monocytogenes*, *Staphylococcus aureus*, *Enterococcus faecalis* and *Bacillus cereus* were analyzed by Liu et al. [108]. This acid showed a significant antibacterial effect and its MIC values were 20–40 µg/mL and MBC values of 32–52 µg/mL [108]. Wojnicz et al. [109] investigated the effect of asiatic acid and its combination with ciprofloxacin on biofilm formation and eradication of uropathogenic *Escherichia coli* (ATCC 700928) and ten clinical strains. The compound weakly inhibited biofilm formation. However, a significantly better effect was obtained when the acid was used in combination with ciprofloxacin. Likewise, biofilm eradication from urological catheters also showed statistically significant results only when asiatic acid was used in combination with the drug.

## 7. Conclusions

The present review showed data obtained from clinical and experimental studies that provide strong evidence of wound healing activity of *Centella asiatica* and its bioactive constituent, asiatic acid. CAE and Aa act in one or more phases of cutaneous repair process. In general, they display activity in tissue regeneration, cell migration and wound repair process by promoting inhibition of pro-inflammatory mediators release and immune cells migration in injured tissue as well as fibroblast proliferation and extracellular matrix and collagen synthesis. The benefits associated with the antimicrobial activity of this plant and its chemical components should contribute in a complementary way, inhibiting infections during healing. CAE and Aa can also be used for the development of therapeutic medicines with enhanced anti-inflammatory and wound healing activities and better safety profiles to control both acute and chronic wounds.

## Abbreviations

Aa	Asiatic acid
CAE	*Centella asiatica*
ECM	Extracellular matrix
IL	Interleucin
IL-1β	Interleukin-1 Beta
IL-6	Interleukin-6
IL-17A	Interleukin-17A
IL-23	Interleukin-23
NOS	Nitric Oxide Synthase
INF-Υ	Interferon gamma
NF-κB	Nuclear Factor Κb
PDGF	Platelet-derived Growth Factor
ROS	Reactive Oxygen Species
SOD	Superoxide Dismutase
TGF-β1	Transforming Growth Factor Beta
TNF-α	Tumor Necrosis Factor Alpha
VEGF	Vascular Endothelial Growth Factor
